# Enhancing tuberculosis treatment adherence and motivation through gamified real-time mobile app utilization: a single-arm intervention study

**DOI:** 10.1186/s12889-023-17561-z

**Published:** 2024-01-22

**Authors:** Siti Aishah Abas, Nurhuda Ismail, Yuslina Zakaria, Siti Munira Yasin, Khalid Ibrahim, Ismassabah Ismail, Asmah Razali, Mas Ahmad Sherzkawi, Norliza Ahmad

**Affiliations:** 1 Department of Public Health Medicine, Faculty of Medicine, Universiti Teknologi MARA Sungai Buloh Campus, Sungai Buloh, Selangor 47000 Malaysia; 2 Department of Pharmaceutical Life Sciences, Faculty of Pharmacy, Universiti Teknologi MARA Puncak Alam Campus, Puncak Alam, Selangor 42300 Malaysia; 3 Centre of Foundation Studies, Universiti Teknologi MARA Cawangan Selangor, Kampus Dengkil, Dengkil, Selangor 43800 Malaysia; 4 Disease Control Division, Sector TB/Leprosy, Ministry of Health, Putrajaya, 62590 Malaysia; 5 TB/Leprosy Disease Unit, Selangor State Health Department, Seksyen 9, Shah Alam, Selangor 40100 Malaysia; 6 TB/Leprosy Disease Unit, Negeri Sembilan State Health Department, Jalan Rasah, Bukit Rasah, Negeri Sembilan, Seremban, 70300 Malaysia

**Keywords:** Video direct observe therapy (VDOT), Mobile health (mHealth), Tuberculosis, Medication adherence, Mobile application, Gamification

## Abstract

**Background:**

Finding innovative methods to enhance Tuberculosis treatment adherence in Malaysia is imperative, given the rising trend of non-adhere TB patients. Direct Observed Therapy (DOTS) has been used to ensure Tuberculosis (TB) drug compliance worldwide. However, due to its inconvenience, digitalizing this system into a virtual monitoring system via a mobile app can help deliver a more efficient tuberculosis management system. A gamified video-observed therapy is developed that connects three users the patient, supervisor, and administrator, allowing drug monitoring and patient loss to follow up with the patient tracking system. Thus, the objective of this study is to determine the impact of Gamified Real-time Video Observed Therapy (GRVOTS) mobile apps on patient medication adherence rates and motivation.

**Methods:**

71 patients from 18 facilities participated in the 8-week single-arm intervention study. GRVOTS mobile apps were installed in their mobile apps, and patients were expected to fulfill tasks such as providing Video Direct Observe Therapy (VDOTS) daily as well as side effect reporting. At 3-time intervals of baseline,1-month, and 2-month intervals, the number of VDOT taken, the Malaysian Medication Adherence Assessment Tool (MyMAAT), and the Intrinsic Motivation Inventory (IMI) questionnaire were collected. One-sample t-test was conducted comparing the VDOT video adherence to the standard rate of 80%. RM ANOVA was used to analyze any significant differences in MyMAAT and IMI scores across three-time intervals.

**Results:**

This study involved 71 numbers of patients from 18 healthcare facilities who showed a significantly higher treatment adherence score of 90.87% than a standard score of 80% with a mean difference of 10.87(95% CI: 7.29,14.46; p < 0.001). The participants’ MyMAAT and IMI scores significantly increased over 3-time intervals with the IMI Interest domain showing the highest mean difference 19.76 (95% CI: 16.37, 21.152: p < 0.001).

**Conclusions:**

By utilizing GRVOTS, a mobile application based on gamification and real-time features, we can enhance motivation and medication adherence among TB patients, while also addressing the limitations of physical DOTS.

**Trial registration:**

IRCT20230308057657N1, Registered on (15/03/23).

## Background

Tuberculosis has stood as the most concerning global infectious disease for three decades, with over 10 million new cases reported annually worldwide [1]. According to a model projection by Ismail N. et al. (2013), the observed and projected TB incidence cases in Malaysia from 1990 will reach up to 30,000 cases in 2030 [2]. After being diagnosed with Tuberculosis, patients are required to successfully finish a 6-month medication regimen utilizing Direct Observed Therapy Short Course (DOTS), a well-established method recognized globally for enhancing medication adherence in TB programs. Nevertheless, despite the introduction of physical DOTS in 1994, Malaysia has encountered persistent challenges with TB loss to follow-up prevelance fluctuating between 4.0% (2010) and 4.8% (2015), and more recently surging to 5.6%, as indicated by the most recent research data [3].

Mobile health technology offers significant potential to improve the management of chronic infectious diseases like tuberculosis (TB). It addresses the critical challenge of ensuring patient adherence to complete treatment courses, vital in preventing TB spread and drug-resistant strains. By fostering patient empowerment, self-management, and connecting patients with healthcare professionals, mobile health technology enhances disease management for TB and similar chronic infections [4–6].

In 2015, the World Health Organization published a roadmap outlining its strategy to integrate digital health into TB prevention and care, with a key focus on enhancing medication adherence support [7]. Digitalization of TB physical DOTS to VDOT via mobile app systems could help with more efficient service delivery. However, among the few apps developed for use by TB patients, none were designed to support TB patients’ involvement in and management of their care. In a scoping review regarding “The Use of Digital Technology to Enhance Tuberculosis Control”, only three out of 145 studies of health apps look into healthcare providers’ and patients’ perspectives in their analysis [8]. It mainly focused on utilizing digital health technology feedback by health professionals rather than TB patients. One study even called for increased patient support focus after reviewing 24 TB health-related apps and argued that TB patient care apps had minimal functionality, were designed primarily for healthcare workers and were more focused on data collection [9].

Additionally, Abdul Rahim and Thomas (2017) emphasized the potential of gamification in improving treatment adherence, as evidenced in their study on using gamification to encourage medication adherence in epilepsy [10]. Their study demonstrated that a gamified approach to epilepsy treatment not only enhances patient adherence but also has the potential to positively influence public perceptions of the disease if shared with a broader audience. Game dynamics can improve user desire and motivation by establishing rules that encourage users to explore and learn about apps, thus maintaining usage sustainability [11]. With a balance of intrinsic and extrinsic rewards, gamification has the element of a design-thinking focused process to engage and motivate the audience into desired behaviors. Thus, it has the potential to transform dull routine tasks into a more enjoyable and motivating experience and has proven to increase intrinsic motivation [12].

Gamified-Real-Time Video Observed Therapy (GRVOTS) is a mobile application specifically designed to bridge the gap in patient support by incorporating gamification, real-time features, and motivational elements to enhance medication adherence, motivate patients, and ensure continued engagement with the app [13]. This paper aimed to document the concept of GRVOTS mobile apps and report its impact towards patients’ motivation and treatment adherence.

## Methods GRVOTS mobile apps

The GRVOTS mobile app is used by three types of users: patients, supervisors, and administrators, each with their own unique dashboard upon login. Patients see information related to their medication, progress, side effects, and virtual video observations. Supervisors have access to VDOT verification and side effect reporting. Administrators can manage user accounts and view patient lists. Data, including real-time VDOT and adverse effect reports, is collected and accessible to supervisors and the TB management team for clinical interventions. Additionally, there’s a website for supervisors and administrators to monitor patient status and activities, with the development process recently published [13].

### The intervention study -tools and data analysis

A single-arm intervention study involves administering the intervention to a sample of individuals with a specific medical condition and tracking them over time to observe the resulting outcomes [14]. Between September and November 2022, a cross-sectional research approach was used to investigate how patients utilized GRVOTS over two months. Data collection was carried out with two established tools: the Malaysian Medication Adherence Assessment Tool (MyMAAT), which assesses medication adherence habits, and The Intrinsic Motivation Inventory (IMI), a questionnaire based on self-determination theory designed to measure intrinsic motivation levels. Data collection involved the use of a self-administered questionnaire, and the gathered information was recorded in an Excel format. One-sample t-test was conducted comparing the VDOT video adherence to the standard rate of 80%. RM ANOVA was used to analyze any significant differences in MyMAAT and IMI scores across three-time intervals. Data analysis was performed using Excel and exported to the Statistical Package for Social Science Software (SPSS version 28).

### Participants and recruitment

The population of this study included patients selected from the 18 facilities consisting of two government hospitals and another 16 primary health clinics. Eligible patients were those who were able to speak and read English or BM, working-aged TB patients (19–64 years old), newly diagnosed with pulmonary TB (first-line treatment), who completed the in-person DOTS at least two weeks (ensuring medication tolerance), were able to provide informed consent and were knowledgeable in smartphone and app usage. We excluded patients with drug-resistant TB and patients with health conditions that disallow them from using a smartphone (severe arthritis, vision impairment).

Participants who fit the recruitment criteria were offered mobile apps during their clinic visit. The informed consent form clearly explained that study participation was voluntary, and participants could withdraw from the study at any time without jeopardizing their health care. Their decision to withdraw had no impact on their relationship with their treatment center. If they wished to withdraw from study participation, they needed only to inform the principal investigator (PI), and no further data would be collected from that time onward.

**S**ample size estimation was calculated using the pairwise comparison formula. Preliminary data indicate that the control mean was 2.54 (SD:0.89) compared to intervention 3.41 (SD 0.89). If the type 1 error probability and precision are 0.05, we will need to study 55 samples. We managed to recruit 88 patients from 18 health facilities and analyzed 17 patients lost to follow-up.

### Procedure

Each participant was asked to sign a consent form and complete a brief demographic questionnaire, including smartphone experience. The app was then downloaded onto the patient’s android phone. Face-to-face training was provided by the researcher, and the tutorial was provided in the mobile application. Participants were asked to demonstrate the VDOT by taking and sending one video during this process. They were also asked to send and use the side effect reporting system. Participant could obtain technical support throughout the study from the researcher by email or phone. Supervisors need to learn how to complete the task of approving the VDOT and side effect reporting. After the training session, the patient and supervisor need to use the mobile apps to complete the task of sending and approving VDOT as well as side effect reporting every day for 2 months. Before, at the 4-weeks and 8 weeks follow-up, participants completed the MyMAAT and IMI questionnaire.

## Results

### Sociodemography

Eighty-two participants (patients) initially agreed to participate in the GRVOTS. Among them, 71 patients completed two months of trials. 11 (13%) participants were lost to follow-up. A total of 71 eligible participants were selected from 18 healthcare centers. The median age was 38 (interquartile range [IQR] 19–65) years, 64.9% (48/71) were male, and 90.4% (66/71) were Malaysian. Out of 71 participants, 90.1% (64) tested negative for HIV and showed BCG scars, while 62% [15] were non-smokers. Most participants, comprising 71.6% (63 out of 71), are employed, and 60.6% (43 out of 71) of patients have a monthly income exceeding RM2160. This factor holds significance as non employed and lower income levels are associated with loss to follow up [16]. The descriptive table regarding the participants is shown in Table 1 below.

**Table 1:** Descriptive Table Sociodemographic of the participant n = 71

Variable	Median (Range)	Frequency%(N)
Sociodemographic		
Gender Male Female		64.9 (48) 31.1 (23)
Nationality Malaysia Non-Malaysian		90.4(66) 6.8 (5)
Occupation Non employment Employment		24 (17) 76 (54)
Education level No education Primary Secondary Higher		7 (5) 2.8 (2) 69 (49) 21.1 (15)
Household income < 2160 > 2160		39.4 (28) 60.6 (43)
TB Profile		
HIV status HIV Positive HIV Negative Unknown		1.4 (1) 90.1 (64) 8.5 (6)
BCG scar Absent Present		9.9 (7) 90.1 (64)
Smoking Status Non-Smoking Smoking	2500(500–7000) 2398.61(1270.585)	62 (44) 38 (27)

### Descriptive number of videos

Approximately 7% of video clips submitted during the first two months of treatment were corrupted because of a software bug that was subsequently fixed. Despite the corruption, the patient kept the video clip in their gallery and utilized WhatsApp as an alternative platform to share it with their supervisor. If the issue persists for more than three days, the patient will lose eligibility for GRVOTS, necessitating a shift to physical DOTS for ongoing treatment. However, similar occurrences are common in trial studies, as observed in various other research investigations [17]. Thus, in our main analysis, we assumed that the submission of a corrupted clip represented pill ingestion since patients were unaware that their submitted videos were corrupted. Table 2 shows descriptive statistics of the study variables.

**Table 2:** Descriptive table on overall video received or not received n = 71

Variable	(Med/Mean)
Video received	3871
Video not received -failed to be sent	301
Video with technical issue-cannot be viewed	545
Total video expected	4260

### Treatment adherence

As for the treatment adherence rate was calculated based on number of videos received over the total video expected. The t-test of the treatment adherence level indicates good treatment adherence of the participant of GRVOTS as a mobile application as shown in Table 3.

**Table 3:** The one-sample t-test of the treatment adherence rate of all participants

	N	Mean (SE)	Mean paired difference (95% CI)	Ta (df)	p-value
Treatment adherence	71	90.87(1.80)	10.87 (7.29,14.46)	6.07(70)	< 0.001

The results of this study can be categorized into four distinct outcomes: three outcomes pertain to the Intrinsic Motivation Questionnaire (IMI), specifically addressing Interest, Competence, and Choice, while the fourth outcome relates to the MyMAAT score. The comparison of mean percentage scores for the three components of IMI and MyMAAT across three different time intervals yielded three separate sets of results.

### Intervention Results

#### (i) Within-subject difference (time effect)

Overall, there is a significant increase in all scores of IMI and MyMAAT across three-time intervals after using the GRVOTS mobile apps as shown in Table 4. The participants’ adherence score (MyMAAT) and motivation score (IMI) scores significantly increased over 3-time interval. Therefore, the intervention is effective.

**Table 4:** Effect of GRVOTS mobile apps usage on 3 component of IMI marks and MyMAAT Marks

Outcome	Baseline (Mean +- SD)	1st month (Mean +-SD)	2nd month (Mean +- SD)	F-stat (df)	Partial eta square	p-value
MyMaat	49.68(6.05)	50.70(5.83)	55.06(6.17)	15.74 (1,70)	0.184	< 0.001
IMI (Interest)	21.48(5.342)	27.48(8.634)	40.14(6.72)	139.29 (1,70)	0.666	< 0.001
IMI (Competence)	20.28(4.32)	23.08(4.86)	27.79(4.79)	48.52(2,140)	0.409	< 0.001
IMI (Choice)	15.38(3.47)	17.72(5.15)	23.87(5.98)	62.00(1366.51,140)	0.470	< 0.001

Repeated measure ANOVA (time-effect)

(Ho: There is no change of MyMAAT and IMI in 3 repeated measurements)

#### (ii) Within-subject contrast table

Regarding the test within-subject contrast table as shown in Table 5, there was a significant increase in the MyMAAT and IMI score score from T1 to T2 and T0 to T2 with mean of 4.35 (1.78, 6.93) and 5.38 (2.67, 8.10) respectively. However, it’s important to highlight that there was no statistically significant increase observed in the MyMAAT score from T0 to T1 with mean of 1.03 (95% CI: 1.14,3.20). MyMAAT marks showed the lowest mean different from T0 to T2 only increase by 5.38 (95% CI: 2.67, 8.10: p < 0.001). Whereas the IMI Interest domain showing the highest mean difference by 19.76 (95% CI: 16.37, 21.152: p < 0.001).

**Table 5:** Post Hoc Comparison of marks for each pair of time levels

Outcome	MyMaat	p-value	IMI (interest)	p-value	IMI(Competence)	p-value	IMI (Choice	p-value
T0-T1 mean difference (95%CI)	1.03 (1.14, 3.20)	0.746	5.75(2.64, 8.86)	< 0.001	2.80 (0.90, 4.71)	0.002	2.34 (0.40, 4.28)	0.013
T1-T2 mean difference. (95% CI)	4.35 (1.78, 6.93)	< 0.001	13.01(10.09, 15.94)	< 0.001	4.70 (2.77, 6.64)	< 0.001	6.16 (4.16, 8.15)	< 0.001
T0-T2 mean difference. (95% CI)	5.38 (2.67, 8.10)	< 0.001	18.76 (16.37, 21.15)	< 0.001	7.51 (5.69, 9.33)	< 0.001	8.49 (6.63, 10.4)	< 0.001

Repeated measure ANOVA (time-effect)

(Ho: There is no change of MyMAAT and IMI in 3 repeated measurements)

#### (iii) Contrast (Post Hoc Comparison)

**Fig. 1:**
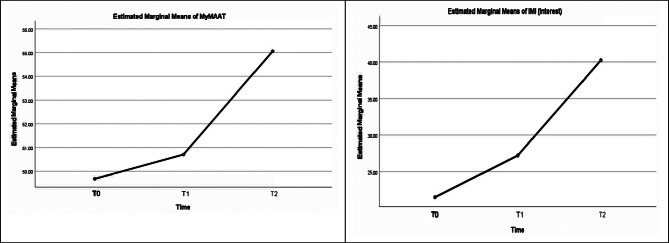
Profile plot of estimated marginal means of MyMAAT (Left) and IMI (Interest) (Right)

**Fig. 2:**
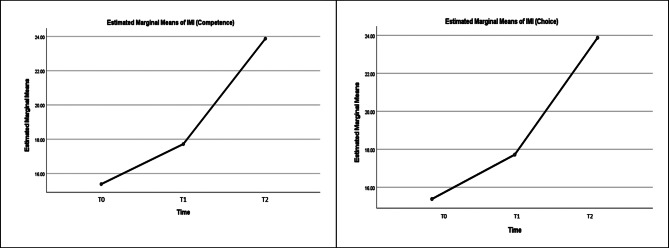
Profile plot of estimated marginal means of My IMI (Competence) (Left) and IMI (Choice) (Right)

Profile plot lines of intervention illustrate the improvement in the mean score of IMI and MyMAAT throughout the 3-time intervals (Figs. 1 and 2). The post hoc comparison of MyMAAT and IMI (Competence) shows a linear trend by (F (1,70) = 23.63, p < 0.01, partial eta = 0.252 (> 0.15), power = 0.998 and by (F (1,70) = 102.306), p < 0.01, partial eta = 0.594 (> 0.15), power = 100 (> 100%) implying that there is a consistent increase in the response variable (MyMAAT score and IMI (Competences) score) across the three time periods.

Whereas the post hoc comparison of IMI (Interest) and IMI (Choice) showed quadratic trend by F (1,70 = 10.34), p = 0.002, partial eta 0.129 = (> 0.15), power = 0.887 (> 100%) and by F(1,70) = 7.333, p = 0.009, partial eta 0.095 (> 0.15), power = 0.761). The quadratic trend scores demonstrate a significant non-linear pattern over time. Assuming a positive quadratic trend (concave up), the intervention initially led to a slow change in scores, followed by acceleration, a peak between measurement points, and eventual leveling off or a slight decrease. Overall, the intervention had a meaningful impact on participants’ IMI (Interest) and IMI (Choice) scores, showing a gradual and increasingly positive effect initially and speed upafter one month of intervention.

## Discussion

Discovering an innovative approach to enhance TB medication adherence is of utmost importance, and implementing a mobile app-based VDOT system with gamification and real-time elements to foster motivation could potentially offer a viable solution.Gamification, defined as applying gaming principles to non-game activities to influence behavior with the potential to enhance engagement and encourage desired actions, such as task completion [18–21]. Mobile apps also hold promise in improving chronic disease self-management [22]. In GRVOTS mobile apps, gamification is integrated to enhance user experience and sustainability [13]. This study aims to assess the GRVOTS mobile app’s impact on patient treatment adherence and motivation.

### Treatment adherence rate

According to various guidelines, maintaining a high level of adherence at 80% or above over two weeks is a positive and statistically significant achievement [23, 24]. In this study, the average treatment adherence score was 90.87% (1.796), which was significantly higher than the standard treatment adherence score of 80% with a difference of 10.873 (95% CI 7.29, 14.46; p < 0.001). The research findings align with previous studies that have supported the use of mobile phones to improve medication adherence among tuberculosis patients or users. Similar adherence levels have been reported throughout high and middle to low-income settings where VDOTS is utilized, including the United States countries [25–28]. Our adherence rate was higher than the median adherence rates reported in studies conducted in Vietnam, Kenya, Uganda, and the USA [29–31]. Moreover, our study demonstrated a treatment adherence rate of 90.87% through GRVOTS, significantly surpassing the Malaysian TB treatment adherence rate of 81% recorded in 2017 via physical Directly Observed Therapy Short Course (DOTS) [32].

### Intervention effect of RM ANOVA in MyMAAT (medication adherence score)

This study found a significant improvement in the patient’s medication adherence score (MyMAAT) with the GRVOTS mobile apps throughout the 3-time interval. The time-intervention effect produces a significant difference in the mean percentage score of MyMAAT score time between pre- and 1 to 2 months post-usage of GRVOTS with significant positive liner trend indicating that GRVOTS positively impacts medication adherence, especially among working-age Tuberculosis patients consistently.

GRVOTS mobile apps have been found to enhance medication adherence, as supported by Wiecek et al.‘s (2020) study, which demonstrated that using a digital therapeutic mobile app can maintain optimal adherence rates in adults with chronic conditions for an extended period [33]. In addition, Tran et al.‘s (2022) scoping review also demonstrates that mobile apps incorporating gamification, reminders, incentives, education, and social community interventions positively impact long-term medication adherence, highlighting the significant promise of integrating mobile technology for effective management in practical settings [34]. The review highlights mobile technology’s promise in enhancing real-world medication adherence, emphasizing its significant potential for effective management through integrated features like gamification, reminders, incentives, education, and social community interventions.

As for the implication from the trend, after post-hoc analysis, it is shown to be linear in trend which means constant small increase in MyMAAT score over time. It could be attributed to the habit of medication intake, which typically takes about 21 days or more to establish. Therefore, it might take more time to observe a significant difference in MyMAAT results compared to our 2-month intervention study [35].

### Intervention effect of RM ANOVA in IMI (Instrinsic Motivation score)

This study found a significant improvement in patients with Tuberculosis disease motivation across subdomains of interest, competence, and choice after using the GRVOTS mobile apps throughout the 3-time interval. The time-intervention effect produces a significant difference in the mean percentage of IMI score of time between pre- and 1 to 2 months post-usage of GRVOTS, indicating that GRVOTS can increase patient motivation, especially among working-age tuberculosis patients. The findings align with many studies supporting that mobile phone apps can improve user motivation.In a study conducted by Jeno, Grytnes et al. (2017), the relationship between achievement scores, perceived competence, and intrinsic motivation in mobile app learning (specifically ArtsApp) was examined and compared to traditional book learning. The findings revealed a significant association between ArtsApp mobile app usage and achievement, with intrinsic motivation mediating this relationship [36].

Mobile apps containing gamification features for medication adherence have shown promising results in improving medication adherence via motivation among users. According to one scoping review regarding “The Use of Gamification and Incentives in Mobile Health Apps to Improve Medication Adherence”, it was found that gamification features in mobile applications, such as dosage reminders, incentives, education, and social community interventions in mobile apps, can contribute to maintaining optimal medication adherence over time [34]. Furthermore, game elements and app features such as rewards can be used as tools to support basic psychological needs that align with the self-determination theory of Desi and Ryan for behavior change in various health areas such as medication adherence [37]. Overall, gamification features in mobile applications can improve medication adherence among users by fostering motivation and resilience and incentivizing the user’s need to fight their illnesses.

As for the implication from the trend, out of the three motivation components measured, the subdomains of interest and choice showed significant quadratic trend increases across the three-time intervals. The increment indicates that the intervention had a gradual and increasingly beneficial effect on IMI scores, with the most significant changes occurring at T1 to T2 after the first month of mobile app usage. In other words, the gamification component in mobile apps was much appreciated and effective after a specific duration of usage, which was one month.

There are a few reasons for the drastic increment: first: in our gamified mobile apps, the progression mechanism, such as the progress meter, performance progress, color change calendar, and badge reward, unlocked and advanced significantly only after using the app for a month. Therefore, after a month, only patients would feel most of the app’s satisfaction effect, and this intrinsic motivation subdomain will improve user engagement and motivation [38].

Secondly, different users have different learning styles; some may take up to 3 months to become familiar with certain mobile apps. Users may need time to become familiar with the app’s gamification features, understand the available choices and options, and learn how their choices impact their experience. As they become more experienced and knowledgeable about the app’s gamified elements, they may feel more confident in making choices that align with their interests and preferences, subsequently increasing the interest and choice component [39].

Thirdly, app developers often iterate and improve their gamification systems based on user feedback and data analysis. After a month of usage, users may notice refinements and updates that enhance the interest and choice component. Our study asked some patients to update their mobile app version to cater to a smoother VDOT feature. These improved apps provide more tailored recommendations based on user preferences and behaviors, increasing users’ ability to make choices aligned with their interests. As users become more familiar with the app, progress in their gameplay, personalize their experience, engage socially, and receive feedback and improvements, mobile app gamification’s interest and choice component is likely to increase significazntly. It can result in a more engaging and motivating user experience over time [37].

Out of all three subdomains, only competence showed a linear but not quadratic trend, which means that throughout the intervention, the motivation level of competence gradually and constantly increased. The competence component of mobile app gamification can constantly and gradually increase after intervention via several components of competency which are skills and gamification elements implemented.

Users can improve their skills by engaging with the app’s gamified features. As they become more familiar with the app and gain experience, their perceived competence gradually increases [40]. This skill improvement happens through encountering various challenges and tasks within the app’s gamified environment. By actively participating and successfully completing these tasks, users gain a sense of accomplishment and reinforce their belief in their own competence. The app’s design is important for enhancing users’ skills, including clear progression, goals, and feedback. Tutorials, exercises, and resources can also support users in acquiring new competencies. As users witness their progress, their confidence grows, motivating them to continue using the app and improving their skills. Active engagement and practice contribute to skill improvement and sustained engagement with the app’s gamified features [38].

Gamification in mobile apps has been found to enhance users’ competence domain in intrinsic motivation. The design and user experience of gamified apps play a crucial role in this increase. Immersion-related elements in app design fulfill users’ needs for competence, autonomy, and relatedness, thereby boosting intrinsic motivation. Effective gamification interventions provide a sense of progression and challenging tasks, motivating users to enhance their skills and overcome new challenges. Timely and meaningful rewards, along with feedback and recognition, validate users’ growing abilities and contribute to increased competence [41]. Goal-setting mechanisms allow users to set specific targets and break them down into achievable tasks, fostering a sense of mastery. As users achieve their goals, their perceived competence improves, further enhancing the competence component. As whole, gamified mobile app interventions that incorporate skill development, clear progression, feedback and recognition, goal setting, and social comparison can lead to a gradual and consistent increase in the competence component. This, in turn, drives users’ motivation, engagement, and overall skill improvement [42].

Out of all three components of intrinsic motivation, the interest component has the highest mean difference marks of T0 and T2 by 18.76, followed by choice and competence. The highest mean difference is due to the interest component being the most significant component of the intrinsic motivation inventory [43]. When individuals are interested in an activity, they are more likely to engage in it willingly and persistently, even in the face of challenges. Interest can also enhance individuals’ perceived competence, which in turn can further reinforce their intrinsic motivation [42].

The significance of this finding is twofold. First, it highlights the importance of fostering interest in promoting intrinsic motivation. You can enhance their motivation and engagement levels by designing activities, tasks, or learning experiences that tap into individuals’ interests and passions [44].

Second, the significance of the interest component within the intrinsic motivation inventory reinforces its role as a primary driver of individuals’ motivation. Recognizing the impact of interest can help researchers, educators, and practitioners better understand and support intrinsic motivation in various domains, such as education, work, or personal hobbies [15].

#### Limitations and strenght

The single-arm intervention study’s design may present difficulties in evaluating treatment effectiveness. Moreover, it’s important to note that the study’s results may have limited applicability to all Malaysian TB patients due to the exclusive focus on government facilities. This study stands out as high-quality due to its strong methodological design. Unlike many mobile apps, which often lack a solid theoretical foundation, our research included a validation study to confirm the intended elements in the apps. Furthermore, while most studies only focus on treatment adherence, this study also investigates the apps’ capacity to enhance patient motivation [45]. In addition, this study utilized validated tools tailored to the target population, achieved a follow-up rate exceeding 80%, and administered intervention packages under the supervision of medical professionals.

#### Suggested improvement

A community-based trial will be performed to measur the effectiveness of the mobile app intervention toward motivation. A better study design of multiple-arm studies should be performed, as we can compare multiple interventions or doses of the same intervention to a control group to better evaluate the effectiveness of VDOTs against the current standard of care or alternative technologies in resource-limited, high-disease-burden settings. This approach allows for comparing outcomes between the two groups, reducing the risk of bias, and increasing the validity of the results.

In the future, to ensure that this intervention is effective, selecting an appropriate candidate for VDOTS intervention is essential. This is because patient completion rates highly depend on their views about the treatment’s effectiveness and the ailment itself. Research shows a unique link between the belief in the significance of adherence to treatment and the completion rates of TB treatment. People who dropped out of the program were less likely to believe in the treatment, and patients’ apprehension about the reduced efficacy of treatment acted as a driving force for them to adhere to the treatment schedule [46]. Thus, promoting a better understanding of the disease and treatments through health education may be beneficial in raising completion rates [28, 47].

Future studies addressing cost and cost-effectiveness are also needed. In addition, in other settings, such as the United States, VDOTS has successfully been coupled with individualized case management to allow real-time intervention after missed doses; the role of this approach in our setting is unknown and should be explored [48].

Our study’s results reveal a quadratic trend, showing a notable surge in the metric after the first month, followed by stabilization. We suggest extending the study for a 6-month period to gain deeper insights into the motivation pattern. Furthermore, we propose supplementing the trend analysis with a stratified analysis to examine motivation variations among different sociodemographic groups. This approach, considering variables such as age and gender, would enhance the comprehensiveness of the study, as outcomes might diverge based on these factors.

## Conclusion

The GRVOTS mobile app demonstrates effectiveness in not only enhancing treatment adherence with a significantly higher score of 90.87% compared to the standard 80%, but also in elevating patient motivation across three time intervals. In summary, GRVOTS, a theory-based mobile application integrating real-time gamification, stands as a promising innovation for boosting medication adherence and motivation among TB patients. By linking patients, supervisors, and TB administrators, this app offers a potential solution for enhancing the efficiency of TB management systems, including swift defaulter tracing.

## Data Availability

All data supporting the study findings are within the manuscript. Additional details information and raw data are available from the corresponding author on reasonable request.
